# Dissonance encoding in human inferior colliculus covaries with individual differences in dislike of dissonant music

**DOI:** 10.1038/s41598-017-06105-2

**Published:** 2017-07-18

**Authors:** Seung-Goo Kim, Jöran Lepsien, Thomas Hans Fritz, Toralf Mildner, Karsten Mueller

**Affiliations:** 1 Max Planck Institute for Human Cognitive and Brain Sciences, Leipzig, Germany; 2 Institute for Psychoacoustics and Electronic Music, University of Ghent, Ghent, Belgium

**Keywords:** Cortex, Midbrain, Perception

## Abstract

Harmony is one of the most fundamental elements of music that evokes emotional response. The inferior colliculus (IC) has been known to detect poor agreement of harmonics of sound, that is, dissonance. Electrophysiological evidence has implicated a relationship between a sustained auditory response mainly from the brainstem and unpleasant emotion induced by dissonant harmony. Interestingly, an individual’s dislike of dissonant harmony of an individual correlated with a reduced sustained auditory response. In the current paper, we report novel evidence based on functional magnetic resonance imaging (fMRI) for such a relationship between individual variability in dislike of dissonance and the IC activation. Furthermore, for the first time, we show how dissonant harmony modulates functional connectivity of the IC and its association with behaviourally reported unpleasantness. The current findings support important contributions of low level auditory processing and corticofugal interaction in musical harmony preference.

## Introduction

Harmony is one of the most fundamental elements of music. Consonant harmony (i.e., high agreement of harmonic series of a complex sound) is perceived as pleasant whereas dissonant harmony (i.e., poor agreement of harmonics) is perceived as unpleasant in the general population, even with no prior exposure to Western polyphonic music^1^. Throughout the human auditory stream, encoding of dissonance is believed to occur at a very low level, particularly in the inferior colliculus (IC)^2, 3^. Structural and functional features of the human IC have been associated with perception of dissonant harmony. Structurally, intersubject covariance between the regional grey matter volume in the human IC and subjective perception of unpleasantness was reported, suggesting that IC neurons might play an important role in emotional response to dissonance^4^. Functionally, a sustained auditory response mainly from brainstem to prolonged tonal sound, namely “frequency-following response (FFR)”^5^, was found to correlate with the subjective preference for consonant over dissonant intervals based on scalp electroencephalography (EEG) data^6^. Together, these studies are consistent with the idea that emotional responses, musical preferences, and perhaps even the foundation of musical systems are rooted, at least to some extent, in low level auditory processing such as neural computation in the IC^2^. Previous neuroimaging studies focused on investigating a group level effect of dissonant music that is common to all participants^7, 8^, but neuroimaging data showing how well individual variability in IC activation covaries with individual variability in emotional response to dissonant harmony is yet scarce.

In the current paper, we addressed a relationship between individual variability in neuroimaging data and that in behavioural data, in response to dissonant harmony. To this end, we adopted fMRI data from our previous experiment^9^. Twenty-three healthy participants were scanned using a 3 T MR system while listening to 30-s excerpts from instrumental music (e.g., classical piano concerto, big band swing). After each excerpt, participants rated the perceived pleasantness (1 = very pleasant, 2 = pleasant, 3 = unpleasant, 4 = very unpleasant) during a silence period of 6 s. An fMRI volume covering the ventral half of the brain was acquired every second. The original design of the experiment manipulated stimuli in a 2 × 2 factorial design of play direction (forward vs. backward) and harmony (consonant vs. dissonant)^9^. In the present study, we only focused on the contrast between the original music (i.e., “consonant”) and its dissonant counterpart (i.e., “dissonant”). Both excerpts were played in the original direction. To create a dissonant excerpt, the original excerpt was transposed by dissonant intervals and mixed together, resulting in dissonant harmony throughout the whole excerpt in addition to artistic use of dissonant harmony in the original music.

The aims of the current study were twofold:

1. To examine whether individual variability in unpleasantness rating is related to that in BOLD signal. We defined individual variability in unpleasantness rating as an average rating of an original (consonant) musical excerpt subtracted from its dissonant counterpart over 20 musical excerpts for each participant. That is,
  $${\rm{\Delta }}Rating=\frac{1}{20}\sum _{i=1}^{20}(rating({diss}_{i})-rating({cons}_{i}))$$
  where *diss*_i_ notes a dissonant version of the i-th musical excerpt, *cons*_*i*_ notes its consonant version, and *rating*(·) is a participant’s rating of a given stimulus. The reason was that a participant’s rating of dissonant music alone is a mixture of dislike of dissonance and one’s musical preference that is irrelevant to harmony. Because we used the same set of music for all participants^9^, it is possible that one participant might have liked certain musical excerpt (in its original form) more than another participant, and it was actually true in the real data strongly motivating use of this differential index (see Results). Therefore, we used the difference in subjective rating of unpleasantness (“rating contrast”) as an index of how much a person dislikes dissonant harmony while controlling nuisance variability in personal musical preference that is not related to harmony.
  This allowed us to probe individual variability that was not examined in our previous analysis of the same data^9^. In the previous study, a BOLD response to certain music was related to a corresponding pleasantness rating in order to study dynamics of a reward system (i.e., ventral striatum) in response to music^9^. In the current study, we sought a neural signature that covaries with individual differences in preference for harmony. Thus we examined a correlation between the rating contrast and “BOLD contrast” (i.e., a contrast map for the effect of dissonance for each participant).
2. To examine if a similar relationship could be found between the individual variability in functional connectivity and that in subjective rating. To analyse functional connectivity, we used psychophysiological interaction (PPI) analysis and cross-correlation analysis. PPI has been used to test whether a condition modulates functional connectivity^10^. Cross-correlation is sensitive to lagged coupling between distant brain regions as recent fMRI studies have suggested neural relevance of the slow dynamics in BOLD signal^11–19^. By the same logic, we correlated the effect of dissonance in cross-correlation with that in subjective rating.

We hypothesised that (1) BOLD contrast in the IC would be correlated with rating contrast given that previous studies showed that the morphology^4^ and activity^6^ of the IC negatively correlated with unpleasantness. We also hypothesised that (2) the effect of dissonance in functional connectivity throughout the auditory stream would covary with the rating contrast.

## Results

### Stimulus and behaviour characteristics

**Figure 1:**
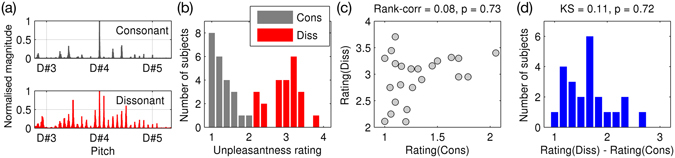
Consonant and dissonant musical excerpts and the subjective rating of unpleasantness by participants. (**a**) Periodograms of an example excerpt (from “Prelude and Fugue No. 3” by J. S. Bach) in its original (i.e., consonant; grey) and dissonant (red) versions. Normalised magnitude is plotted over a log-linear scale of pitch (c.f., D#4 = 311.13 Hz). (**b**) A histogram of subjective ratings of consonant (grey) and dissonant (red) music. (**c**) A scatterplot of ratings of dissonant music over ratings of consonant music. Spearman rank correlation test statistics are noted at the top of the scatterplot. (**d**) A histogram of increased unpleasantness rating due to dissonance (i.e., dissonant – consonant). The Kolmogorov-Smirnov test statistics are shown at the top of the histogram. Abbreviations: cons, consonant; diss, dissonant.

To show characteristics of the musical stimuli and subjective ratings, spectra of the excerpts and histograms of ratings are plotted in Fig. 1. By our manipulation, the periodograms of dissonant music showed dispersed small peaks (i.e., unresolved harmonic components) unlike the original (i.e., consonant) music (Fig. 1a).

Interestingly, the subjective rating of the original excerpts was not constant but varied over participants (Fig. 1b). The ratings were between 1 (very pleasant) and 2.05 (pleasant). That is, the subjective rating reflects individual musical preference already in the absence of any manipulation of the original music. More interestingly, a participant’s rating of dissonant music (between 2.11 and 3.71) was not significantly related to rating of consonant music (Fig. 1c; Spearman rank correlation r = 0.08, p = 0.74). This indicates that how much one likes the original music alone cannot predict how much the one would like the dissonant music. Therefore we used the effect of dissonance in unpleasantness ratings as a dissonance-dislike index, namely “rating contrast” in the following analyses. The distribution of the rating contrast was very similar to the Gaussian distribution (Fig. 1d; Kolmogorov-Smirnov test statistic = 0.11, p = 0.72).

### Functional activation analysis

**Figure 2:**
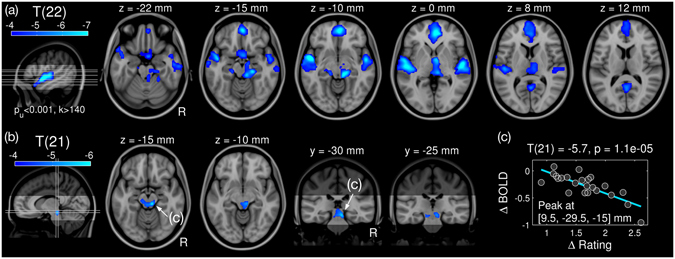
Effect of dissonance in functional activation. (**a**) T-map (d.f. = 22) for the effect of dissonance is shown in axial slices with MNI152 coordinates above each slice. Locations of axial slices are marked as a grey line in the left most sagittal slice. Anatomical image is shaded to highlight the actual slab of fMRI scans. (**b**) T-map (d.f. = 21) for intersubject correlation between the BOLD contrast and rating contrast. Axial and coronal slices are shown also with coordinates. (**c**) Scatterplot of BOLD contrast over rating contrast with a regression line (cyan) at the peak voxel (marked by arrows in (**b**) z = −15 mm; y = −30 mm) for the intersubject correlation. Family-wise error rate (FWER) was controlled to be less than 0.05 by cluster-extent thresholding. Abbreviation: d.f., degrees of freedom.

Previous fMRI studies reporting a wide range of brain regions showed reduced BOLD activation in response to dissonant music compared to consonant music^7, 8^. To illustrate the effect of dissonance that we correlated with the rating contrast, we tested a common effect of dissonance in BOLD activation by a group-level general linear model (GLM) as:

1 $${\rm{\Delta }}BOLD={\beta }_{0}+error,$$

where Δ*BOLD* (or “BOLD contrast”) is the subject-level BOLD contrast between the dissonant and consonant music, *β*_0_ is an unknown parameter to estimate, and error is Gaussian noise. All cluster-wise p-values were corrected for multiple comparisons correction (see Methods). As expected, we found significantly decreased BOLD signals in a number of clusters (Fig. 2a) including the brainstem, ventromedial prefrontal cortex (vmPFC), precuneus, and the bilateral superior temporal gyri (STGs). The found effect is similar to the main effect of dissonance (averaging forward and backward conditions) reported in our previous study^9^, but in this study, we analysed the effect of dissonance without any influence of reversing the play direction. Statistical details of the significant clusters are listed in Table 1a.

**Table 1:** Statistics of the significant clusters with the effect of dissonance. Significance of a cluster was determined by an extent threshold that controls the family-wise error rate (FWER) less than 0.05 (see Methods). (a) Common effect of dissonance in BOLD signal, (b) intersubject correlation between the rating contrast and the BOLD contrasts, (c) common effect of dissonance in psychophysiological interaction (PPI) between the psychological contrast of dissonance and inferior colliculus activity. Identification of anatomical structures was based on Harvard–Oxford cortical/subcortical structural atlases provided in FSL (https://fsl.fmrib.ox.ac.uk/). Abbreviations: BOLD, blood-oxygen-level dependent signal; d.f., degrees of freedom.

Main structure of cluster	MNI-coord. (mm)	Max T	Max Z	Min P (cluster)	Effect Size (β)	Cluster size (cm^3^)
(a) Common effect in BOLD		(d.f. = 22)				
Left superior temporal gyrus	−53, −2, −8	10.54	6.23	<10^−9^	−0.716	17.2
Right Heschl’s gyrus	54, −12, 0	8.51	5.60	<10^−7^	−0.539	12.3
Right paracingulate gyrus	7, 53, −5	8.41	5.57	<10^−9^	−0.684	16.1
Brainstem	10, −32, −8	7.96	5.41	<10^−9^	−0.428	18.0
Subcallosal cortex	4, 3, −20	6.60	4.85	0.015	−0.307	2.2
Right Precuneous Cortex	2, −57, 10	5.96	4.55	0.003	−0.693	3.1
Left crus I of cerebella	−38, −50, −38	5.36	4.24	0.001	−0.212	5.9
(b) Intersubject correlation in BOLD		(d.f. = 21)				
Right inferior colliculus	10, −30, −15	5.72	4.39	0.011	−0.400	2.36
(c) Common effect in PPI		(d.f. = 22)				
Left temporal pole	−56, 10, −18	8.06	5.44	<10^−7^	−0.120	11.1

Then we tested whether this BOLD contrast covaries with the rating contrast using a group-level GLM as:

2 $${\rm{\Delta }}BOLD={\beta }_{0}+{\beta }_{1}{\rm{\Delta }}Rating+error,$$

where *β*_0_, *β*_1_ are unknown parameters to estimate. Strikingly, we found a strong negative correlation between the BOLD contrast and the rating contrast in the bilateral ICs with a cluster peak in the right IC (Fig. 2b). That is, the more a participant disliked dissonant harmony, the more IC activation was decreased by dissonance. To illustrate fitting of regression and distribution of datapoints, a scatterplot with a regression line at the peak voxel in the right IC is given in Fig. 2c. Statistical details of the significant cluster are listed in Table 1b.

### Psychophysiological interaction analysis

**Figure 3:**
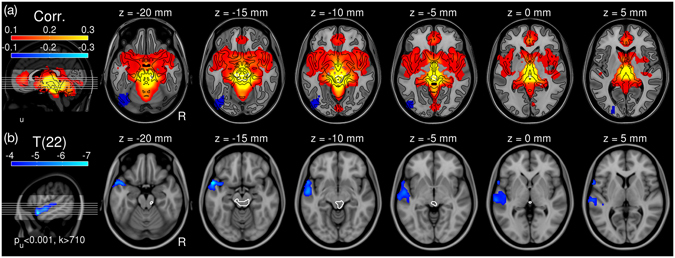
Functional connectivity of the right interior colliculus (IC). (**a**) Pearson’s correlation map seeding from the IC. For visualisation, tissue boundaries are marked in black contours. (**b**) T-map (d.f. = 22) for psychophysiological interaction between the psychological contrast (dissonant vs. consonant music) and the BOLD time series in the IC, which is marked in white. Family-wise error rate (FWER) was controlled to be less than 0.05 by cluster-extent thresholding. Abbreviation: d.f., degrees of freedom.

We further studied the functional connectivity of the IC cluster (Fig. 2b) using PPI analysis^10^, where we test an interaction between a psychological factor and a physiological factor (see Methods). In the current study, the psychological factor was the contrast of conditions (i.e., dissonant – consonant), of which the main effect is already shown (Fig. 2a). The physiological factor was the first eigenvariate of the BOLD time series from the IC cluster as a region-of-interest (ROI). Its main effect is illustrated by a Pearson’s correlation map (Fig. 3a). The IC showed extensive positive correlations with the limbic and cortical systems including the hippocampi (HCs), parahippocampal gyri (PHC), amygdalae, thalami, ventromedial prefrontal cortex (vmPFC), precuneus, and temporal pole (TP). These cortical regions also showed reduced BOLD activation in response to dissonance in previous neuroimaging studies^7, 8^.

Consequently, we tested an interaction between two factors (i.e., PPI). That is, we tested whether the functional connectivity of the IC (Fig. 3a) is different between conditions. Using the group-level GLM (1) with individual PPI contrast maps, we found a significant reduction in the functional connectivity of the IC while listening to dissonant music compared to consonant music in overall subjects. The decreased functional connectivity was found in an extensive cluster in the anterior part of the STG (aSTG) including the planum polare (PP) and TP in the left hemisphere (Fig. 3b). See Table 1c for statistical details. We also tested the relationship between the PPI contrast and the rating contrast using the group-level GLM (2), but did not find a significant effect of rating contrast (min p = 0.51).

### Cross-correlation analysis

Inspired by recent fMRI studies that demonstrated neural relevance of delayed coupling in BOLD time series^11–22^, we further examined the functional connectivity between the IC and the left aSTG using cross-correlation. Cross-correlation of two time series is calculated by computing correlation between a time series with a shift (or delay) and another time series without shift. Unlike zero-lag correlation (i.e., Pearson’s correlation), cross-correlation differentiates the temporal order of events in time series, which suggests the direction of information transfer (see Methods).

**Figure 4:**
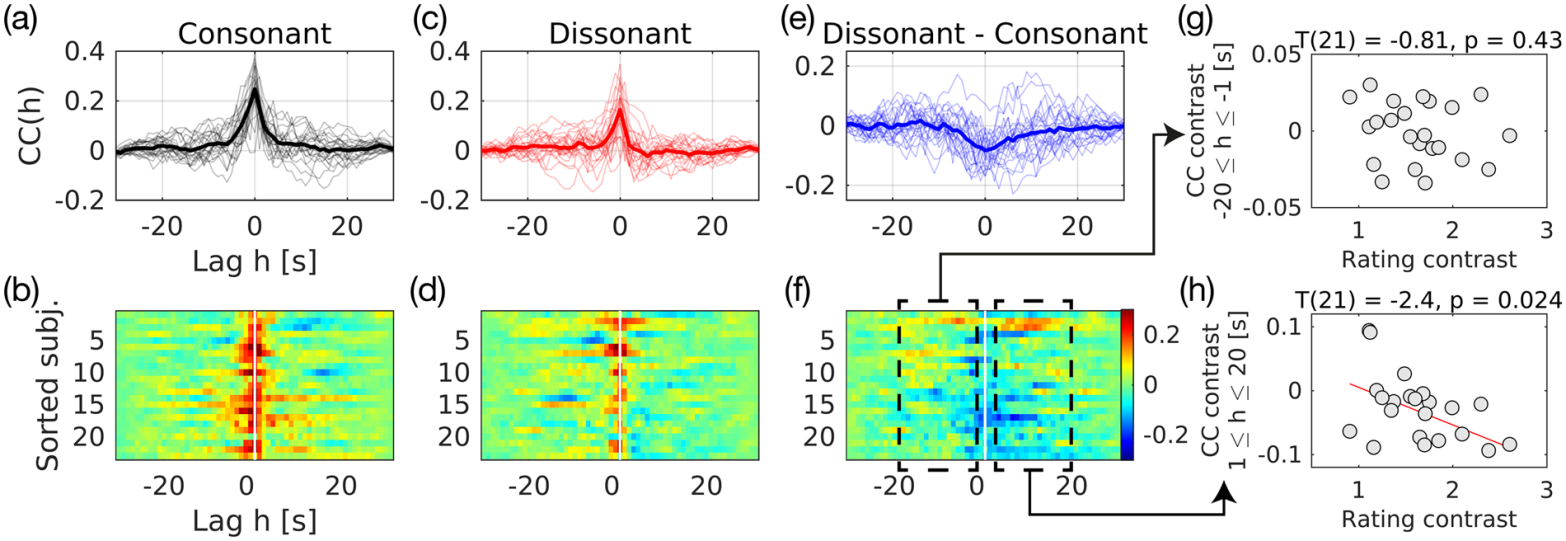
Cross-correlograms for functional connectivity between the right inferior colliculus (IC) and the left anterior aspect of superior temporal gyrus (aSTG). Cross-correlation was computed by shifting the IC time series while listening to consonant (**a,b**) or dissonant (**c,d**) excerpts; a positive lag indicates that the IC time series precedes the aSTG time series with the specific lag. The difference between the consonant and dissonant music is given in (**e,f**). In the overlaid correlograms (**a,c,e**), thin lines show cross-correlations of all individuals and thick lines show averaged cross-correlations. In color-coded correlograms (**b,d,f**), y-axis corresponds to each individual sorted by the unpleasantness rating change with a marking for zero-lag (a white vertical line). We averaged cross-correlation with negative and positive lags separately (dashed black boxes in (**f**)) to relate with a behavioural factor (i.e., “rating contrast”). In the scatterplots (**g,h**), the averaged “cross-correlation contrasts” with negative (**g**) and positive (**h**) time lags are plotted over rating contrast. Abbreviations: CC, cross-correlation; subj., subjects.

The cross-correlation between the IC and the aSTG is shown in Fig. 4. In both conditions, the cross-correlation peaked on average at around the zero-lag (Fig. 4a–d). The difference between cross-correlation functions (i.e., “cross-correlation contrast”; Fig. 4e) was negative at the zero-lag as already seen by the PPI analysis. A novel observation using cross-correlation was that, when sorting the subjects by the rating contrast, the cross-correlation with positive lags (h > 0) appeared to decrease in subjects with higher dislike of dissonance (Fig. 4f).

Thus we tested the relationship between cross-correlation contrast and rating contrast. For simplicity, we averaged the cross-correlation function within either negative (−20 ≤ h ≤ −1 s) or positive lags (1 ≤ h ≤ 20 s). Scatterplots of the positive and negative cross-correlation contrasts over the rating contrasts are given in Fig. 4g and h, respectively. We found a significant, negative effect of rating contrast in the positive cross-correlation contrast (p = 0.024; Fig. 4h). That is, the more a participant disliked dissonance, the more the cross-correlation with positive lags was reduced. Contrarily, we did not find any significant effect for negative lags. Top-down communication from the aSTG to the IC may be reflected in cross-correlation with positive lags (see Methods).

## Discussion

In the current paper, we showed (1) decreased BOLD activation due to dissonance in a number of brain regions including the IC, (2) an inter-subject relationship between dislike of dissonance and reduction of activation in the IC, (3) functional connectivity between the IC and the aSTG being decreased by dissonance, and (4) an inter-subject relationship between dislike of dissonance and reduction of delayed functional connectivity between the IC and the aSTG. We discussed relevance and significance of our findings as follows:

1. In accordance with previous studies^4, 6^, we found a decrease of BOLD activation in the IC due to dissonant music. Our knowledge about the earliest encoding of dissonance in IC neurons derives from electrophysiological evidence: from a single-unit recording of IC neurons of a cat, the dissonance of tone-pairs was encoded by rate fluctuation of the IC neurons that was synchronised with the frequency difference between two tones (i.e., beating)^3^. Furthermore, in human EEG data, behaviourally reported perception of consonance was correlated with the agreement between the spectra of sustained auditory response (i.e., FFR) and a theoretical representation of pitch (i.e., a set of natural harmonics for a given fundamental frequency)^2^. That is to say, the agreement (also known as “neural pitch salience”) was weak in dissonant musical intervals because the high order harmonics of two fundamental frequencies interfere with each other^2^. In fact, reduced FFR amplitude in responding to a “detuned (shifting about 0.5 semitone up or down; thus highly dissonant)” note of a major or minor triad (compared to a counterpart in tune) was reported in participants with minimal experience in musical training^23^. Since participants in the current dataset did not report any experience of formal musical training, it is possible that dissonant music reduced the BOLD response in the IC via a modulation of FFR.
2. We found that individual differences in dislike of dissonance correlated with BOLD deactivation in the IC, similarly to a previous EEG study^6^, where a participant who strongly preferred consonant over dissonant intervals showed higher neural pitch salience in the FFR. Given the possible relationship between the FFR and BOLD activation, this finding suggests that one’s strong disfavour for dissonant harmony could be heavily influenced by vivid representation of dissonance in the IC (encoded by decrease in BOLD activation), even if an individual’s tolerance level of dissonance is not very different from others. Interestingly, it has been consistently shown that neural activity of the IC, as characterised by FFR, seems to directly relate to individual variability in auditory learning and perception such as tonal language learning^24, 25^, musical and linguistic pitch processing^26^, auditory scene segregation^27^, and speaker identification from speech in noise^28^. This seems to suggest that the IC is involved in auditory processing resources, which corresponds to the current finding.
  Please note that the found effect of rating contrast in the current study is different from the effect of unpleasantness rating in our previous study^9^ despite apparent similarity. In the previous study^9^, we modelled brain activity to each musical excerpt by the subjective rating to each excerpts within one individual. In the current study, first we averaged brain response to all dissonant excerpts compared to consonant excerpts. Subsequently, we correlated the individual responses with the subjective ratings of all dissonant excerpts compared to consonant excerpts. In other words, the differences between the previous and current studies were whether the level of correlation analysis was at subject level (i.e., within-subject effect) or group level (i.e., between subject effect) and whether the variables were raw or contrasted.
3. Analysis of PPI revealed functional connectivity between the IC and the left aSTG was reduced while listening to dissonant music. In human lesion studies^21, 29^, a crucial role of the anterior temporal lobe in emotional appraisal of dissonant harmony was suggested. More specifically, epileptic patients who received resection of the anterior temporal lobe (including PHC, HC, amygdala, and TP) did not perceive dissonant harmony as unpleasant even though the detection of dissonance was intact^21^. In another lesion study^29^, patients who underwent an anterior mesio-temporal lobe resection (including PHC, HC, amygdala, TP, and aSTG) rated dissonant music more pleasantly compared to a control group, but in this study, detection of dissonance was also impaired in patients^29^. Taken together, functional connectivity between the IC and the aSTG seems to be involved in emotional response to dissonant harmony.
  An interesting question arising from the PPI analysis is laterality. In the current study, the cortical cluster of the PPI was only found in the left hemisphere. Additional PPI analyses with smaller spherical ROIs also showed the significant PPI only in the left hemisphere (see Supplementary Figure S1 and Table S1; although there is a possibility that the spatial smoothness of the images might be too high to fully separate the left and right ICs). This appears to be contrasted to the widely accepted idea of hemispheric specialization based on consistent findings^30–33^: that is, higher sensitivity in temporal modulation in the left auditory cortex and higher sensitivity in spectral modulation in the right auditory cortex. However, the hypothesis and the current finding are not mutually exclusive because the result of the PPI analysis does not imply that the functional connectivity between the right IC and the left aSTG is stronger than other pairs (e.g., right IC and the right aSTG; the left IC and the right aSTG). Instead, the analysis of PPI only suggests a decrease in functional connectivity due to dissonance regardless of their functional connectivity in general. As shown in Fig. 3a, the IC cluster was highly correlated with the bilateral aSTGs, insulae, and limbic system showing that the involvement of the right aSTG in processing musical excerpts is not weaker than the left aSTG.
  The significance of functional connectivity between the right IC and the left aSTG, particularly in music perception, is rarely known. However, low-level involvement (such as the IC) in music perception has been suggested^34^. An fMRI study reported high inter-subject consistency in the IC activity when listening to the original music compared to listening to distorted counterparts^35^. Moreover, another fMRI study suggested that the left STG is involved in “musical semantic memory”^36^. Taken together, we believe our finding of the modulation of the functional connectivity between the aSTG and the IC might suggest a putatively greater involvement in music appreciation.
4. Finally, we found that individual differences in dislike of dissonance correlated with a decrease in cross-correlation between the IC and the left aSTG, averaged over positive lags. While cross-correlation functions during listening to consonant and dissonant music were unimodal (peaking at around zero-lag) and symmetric, the differences between two cross-correlation functions were asymmetric, suggesting directionality in certain components of the interaction. Indeed, we found a significant effect of rating contrast only in cross-correlation contrast within positive (i.e., [1, 20] s) but not within negative (i.e., [−20, −1] s) lags. This is very interesting because the positive sign of the time lag can be interpreted as top-down modulation from the aSTG to the IC^37^. Descending corticofugal connections are well known to be abundant throughout the auditory stream and their important functional role is theorized to be calibration or reorganisation of low-level processing to enhance or diminish cortical processing of auditory information that is associated with biologically salient events (either rewarding or adverse)^38^.

Nonetheless, it should be noted that the causal relationship between the IC and the aSTG was not formally tested in the current study based on fMRI data. Although the temporal resolution of the current data was higher (1 Hz) than in other fMRI studies on music perception (between 0.3 and 0.5 Hz), it is also true that the current temporal resolution was insufficient to differentiate rapid interaction between the brainstem and cortex along the auditory stream. Because of the slow nature of the neurovascular coupling underlying BOLD signal, increasing the sampling rate of the fMRI sequence may not be sufficient to examine the rapid neurodynamics. For instance, functional near-infrared spectroscopy (fNIRS) with a higher sampling rate of 10 Hz also showed similar results to other fMRI studies in the analysis of frequency-dependent resting-state network^15, 39^. Thus, a follow-up M/EEG study with the same experiment protocol could be more useful to investigate the causal relationship^40^ between the auditory brainstem and the auditory cortex in perceiving dissonance and its emotional appraisal.

In conclusion, we report neuroimaging evidence that the activation and functional connectivity of the IC is well associated with an individual’s dislike of dissonant harmony. We believe that the current findings support the notion that the physiological sensitivity to dissonance of the subcortical auditory system and its interaction with auditory cortex play crucial roles in constituting one’s subjective preference in musical harmony.

## Methods

All analyses used data acquired for a previous study^9^. Thus, only necessary details are reported in this section. Please see the previous paper^9^ for further details on experiment and data acquisition.

### Stimulus

Twenty 30-s excerpts from joyful instrumental tunes (all major–minor tonal music) from the last four centuries were selected^4, 9, 41, 42^. The musical excerpts included classical (e.g., J. S. Bach), swing (e.g., Benny Goodman), and tango (e.g., Francisco Canaro). As mentioned above, the original design of the experiment manipulated stimuli in a 2 × 2 factorial design of play direction and dissonance and we analysed the difference between the two conditions (i.e., forward-consonant; forward-dissonant). To create a forward-dissonant excerpt, the original tune was transposed by two semitones (major seconds) up and six semitones (diminished fifths) down, and mixed together, which resulted in dissonant harmony throughout the whole excerpt in addition to artistic use of dissonant harmony in the original music.

Please note that a local level of sensory dissonance changed over time. It is because, in music as a form of art, various levels of dissonance (e.g., use of diatonic/chromatic chords, chords density, articulation, or timbre of instruments) are dynamically used for artistic and aesthetic purposes. Nonetheless, because we averaged BOLDs signal and ratings over the whole set of musical excerpts to compute the contrast values; we believe that the experiment design and the analysis approach in the current study were sufficient and valid to investigate individual differences in perceiving dissonant harmony.

### Experiment and imaging

Twenty-three healthy participants (13 females; mean age 25.9 ± 2.9 years) were scanned while listening to 80 musical excerpts presented in a pseudorandom order at a volume of about 70 dB SPL. Between 30-s musical excerpts, there was a 6-s silent period for in-scanner rating of unpleasantness. Participants pressed a button on a keypad with four buttons that were mapped to ratings, ranging from 1 = very pleasant to 4 = very unpleasant. During a long, single session lasting 48 min, 15 axial slices of echo planar imaging (EPI) were acquired every second (TR = 1 s), with a voxel size of 2.5 × 2.5 × 4 mm^3^ and an inter-slice gap of 0.5 mm covering the ventral half of the brain.

As stated in our earlier publication using the same dataset^9^, the experiment was carried out strictly following guidelines that had been approved by the Ethics Committee of the University of Leipzig. All participants provided written informed consent before the MRI scanning.

### Image processing

All data were processed using SPM8 (Wellcome Trust Centre for Neuroimaging, London, UK). After static-field bias correction, EPI images were spatially normalised into the Montreal Neurology Institute (MNI) template space, resampled in isotropic resolution of 2.5 mm, and smoothed with a 3-D isotropic Gaussian kernel with FWHM of 8 mm. Spatial normalization was visually inspected from unsmoothed EPI images (see Supplementary Figure S2).

To minimise the artefacts of head motion, we used the “anatomical CompCor” approach^43^, which proposes to model non-neural fluctuation in fMRI data by some principal components extracted from white matter (WM) or cerebrospinal fluid (CSF) voxels. Because head motion introduces coherent change in intensity over a large extent, functional connectivity based on fMRI data can be easily inflated even by a slight head motion^44^. Thus, this idea of CompCor^43^ has been widely used especially in controlling such spurious correlation in analysing resting-state functional connectivity^44^. In this study, as proposed by the original paper^43^, we used CompCor regressors both for the task-based analysis and the functional connectivity analysis for consistency. The WM and CSF voxels were defined by tissue probability greater than 99%. Six principal components were selected for the CompCor regressors.

### Functional activation analysis

Within-subject (i.e., the first-level) analysis was performed by an autoregressive GLM^45^ as:

3 $${\bf{r}}=({\bf{X}}\ast h)\cdot {\bf{b}}+AR(1)+\varepsilon .$$

The residual time series **r** is from a GLM that regresses out non-neuronal fluctuation in BOLD signal as:

4 $${\bf{y}}={\bf{Z}}\cdot {\bf{g}}+\varepsilon ,$$

where **y** is a BOLD time series, **Z** is a nuisance matrix, **g** is an unknown coefficient vector, ε is white Gaussian noise. The nuisance matrix **Z** is simply concatenated 6 rigid-body motion parameters, 6 CompCor regressors, and a constant term. Once **g** is estimated, residual time series was computed by **y** = Z·g + *ε*.

Other terms in the model (3) are as follows: **X** is a design matrix, which binarily encodes musical conditions, *h* is a canonical haemodynamic response function (HRF), * is an operator of convolution, **b** is unknown coefficients. AR(1) is a first-order autoregressive function, which is defined at a time point ***t*** as $AR(1)=\omega {y}_{t-1}$ with a temporal dependency of ω. For this autocorrelation (i.e., non-sphericity across timepoints), restricted maximum likelihood (ReML) hyperparameters were estimated and used for whitening data^46^.

Contrast value is a weighted sum of estimated coefficients as $C=\sum {c}_{j}{\hat{\beta }}_{j}={\bf{c}}\cdot \hat{{\bf{b}}}$ where *c*_*j*_ is the j-th element in a row vector of contrast weights **c** and *β*_*j*_ is the j-th element of a column vector of estimated coefficients $\hat{{\bf{b}}}$. We computed BOLD contrast with a contrast vector that contrasting “forward” vs. “consonant” conditions.

T-statistic for a given contrast vector from a GLM is computed as $T=\frac{{\bf{c}}\cdot \hat{{\bf{b}}}}{s/\sqrt{n}}$ where *s* is a sample standard deviation and *n* is the number of data points. T-statistic is related to the contrast value as $T=\frac{C}{s/\sqrt{n}}$.

### Psychophysiological interaction (PPI) analysis

The modulation of functional connectivity was investigated using the analysis of psychophysiological interaction (PPI)^10^, which is a subject-level GLM for the interaction of physiological and psychological factors. That is, the design matrix for PPI (**X**_PPI_) was given as:

5 $${X}_{PPI}=[\begin{array}{ccc}\phi  & {\bf{X}}\cdot {\bf{c}} & \phi \cdot {\bf{X}}\cdot {\bf{c}}\end{array}].$$

where *φ* is a neural activity vector of a given seed region (i.e., physiological factor). **X** is a design matrix from the equation (3), **c** is a contrast vector of interest, and their dot product **X** . **c** is a psychological factor. Thus $\phi \cdot {\bf{X}}\cdot {\bf{c}}$ is the interaction between physiological and psychological factors.

### Cross-correlation analysis

**Figure 5:**
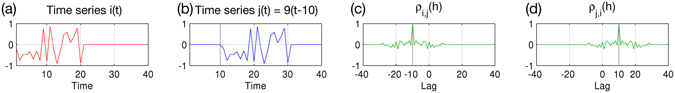
A toy example of cross-correlation. Time series j is a delayed time series i by 10. Therefore cross-correlation (i,j) peaks at the lag of −10 and cross-correlation (j,i) peaks at the lag of 10.

Cross-correlation of two time series *i* and *j* at lag *h* is given by:

6 $${\rho }_{ij}(h)=\frac{{\mathrm{cov}}_{ij}(t+h,t)}{\sqrt{{\mathrm{var}}_{i}(t+h)\cdot {\mathrm{var}}_{j}(t)}}$$

where ${\rho }_{ij}(h)={\rho }_{ji}(-h)$, which is restricted between −1 and 1. The sign of lag implies temporal order to the time series. As given by a toy example (Fig. 5), for an arbitrary time series *i* (Fig. 5a) and its delayed version *j* (i.e., $j(t)=i(t-h)$) with a time lag *h* (Fig. 5b), the cross-correlation between *i* and *j* is one when the lag is *−h* (i.e., ${\rho }_{ij}(-h)=1$; Fig. 5c) and vice versa (i.e., ${\rho }_{ji}(h)=1$; Fig. 5d).

### Statistical inference

The Gaussianness of the subject-level contrasts maps was tested using the Kolmogorov-Smirnov test. False-discovery-rate adjusted^47^ p-values were all 1, which indicates a very high probability of false rejection of the null hypothesis of normality. Thus, we concluded that the Gaussianness assumption holds.

Family-wise error rate (FWER) was controlled less than 0.05 at cluster level using Random Field Theory^48^ as implemented in SPM8. The cluster-forming height-threshold was 0.001 and the extent-threshold was determined by the smallest size of a cluster with a cluster-level p-value less than 0.05 (i.e., two-pass thresholding). Note that the our choice of the cluster-forming threshold was sufficiently high and the extent-threshold was determined not arbitrarily but based on the cluster-level p-values^49, 50^.

## Electronic supplementary material

Supplementary Information
