# Author Correction: Dissonance encoding in human inferior colliculus covaries with individual differences in dislike of dissonant music

**DOI:** 10.1038/s41598-021-01247-w

**Published:** 2021-11-02

**Authors:** Seung-Goo Kim, Jöran Lepsien, Thomas Hans Fritz, Toralf Mildner, Karsten Mueller

**Affiliations:** 1 Max Planck Institute for Human Cognitive and Brain Sciences, Leipzig, Germany; 2 Institute for Psychoacoustics and Electronic Music, University of Ghent, Ghent, Belgium

Correction to: *Scientific Reports* 10.1038/s41598-017-06105-2, published online 18 July 2017

The original version of this Article contains errors in the legend of Figure 4.

“Cross-correlation was computed by shifting the IC time series while listening to consonant (**a,b**) or dissonant (**c,d**) excerpts; a positive lag indicates that the IC time series precedes the aSTG time series with the specific lag.”

should read:

“Cross-correlation was computed by shifting the IC time series while listening to consonant (**a,b**) or dissonant (**c,d**) excerpts; a positive lag indicates that the IC time series follows the aSTG time series with the specific lag.”

