# Supplementary material for: Dissonance encoding in human inferior colliculus covaries with individual differences in dislike of dissonant music
Source: Sci Rep. 2017 Jul 18;7:5726. doi: 10.1038/s41598-017-06105-2 (PMC5516034; doi:10.1038/s41598-017-06105-2)

Supplementary Information

Dissonance encoding in human inferior colliculus covaries with individual differences in dislike of dissonant music

Seung-Goo Kim[1,*], Jöran Lepsien[1], Thomas Hans Fritz[1,2], Toralf Mildner[1], and Karsten Mueller[1]

1 Max Planck Institute for Human Cognitive and Brain Sciences, Leipzig, Germany

2 Institute for Psychoacoustics and Electronic Music, University of Ghent, Ghent, Belgium

* Correspondence should be addressed to S.-G.K.

Postal address: Stephanstrasse 1A, 04103 Leipzig, Germany

Phone: +49 341 9940 2618

Facsimile: +49 341 9940 2624

Email: sol@snu.ac.kr

Psychophysiological interaction with spherical regions-of-interest (ROIs)

In order to confirm the laterality of anterior part of the left superior temporal gyrus (aSTG) that showed significant effect of the PPI (i.e., dissonant – consonant; and time series of ROI). Using the same analysis pipeline as done for the IC cluster as a seed, a significant effect of the PPI was found in the left aSTG with both of the left and right ICs.

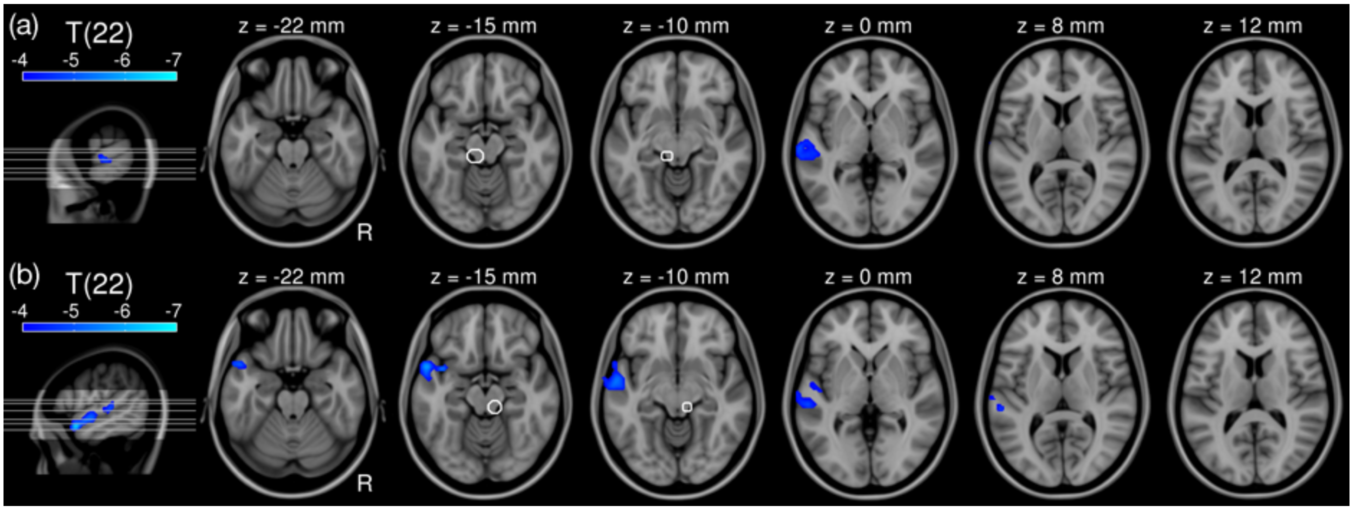

Figure S1. Psychophysical interaction (PPI) from spherical regions-of-interest (ROIs). T-maps (d.f. = 22) for the left (a) or right (b) ROI for the interaction between the psychological factor (dissonant vs. consonant music) and the psychological factor (i.e., BOLD time series in a spherical ROIs of the left or right IC) are shown. The ROIs are marked by white contours. Family-wise error rate (FWER) was controlled to be less than 0.05 by cluster-extent thresholding. Abbreviation: d.f., degrees of freedom.

Main structure of cluster	MNI-coord. (mm)	Max T	Max Z	Min (cluster)	P Effect Size (β)	Cluster size (cm^3)
(a) Left inferior colliculus (-10, -30, -15)		(d.f.=22)				
Left superior temporal gyrus	-66, -22, 0	4.59	3.78	0.010	-0.087	2.09
(b) Right inferior colliculus (10, -30, -15)		(d.f.=22)				
Left temporal pole	-56, 10, -18	6.72	4.86	$<10^{-5}$	-0.095	7.80

Table S1. Statistics of the significant psychophysical interaction (PPI) seeding from (a) the left inferior colliculus (IC) and (b) the right IC. The centres of spherical ROIs with the radius of 7 mm are given in the table. Degrees of freedom is 22 for both tests. Identification of anatomical structures was based on Harvard–Oxford cortical/subcortical structural atlases provided in FSL (https://fsl.fmrib.ox.ac.uk/).

Visual inspection of spatial normalisation quality

Good registration of individual images into template space is critical in following analyses. Thus we showed representative axial slices (z = -10 mm), where we found significant effect of "rating contrast" and "BOLD contrast".

Figure S2. Individual axial sections (z = -10 mm) of EPI images normalised in MNI152 space. The EPI images were averaged over whole section. To aid identification of neuroanatomy of readers, contours at abrupt image intensity change from the MNI152 template of T1-weighted image are superimposed in dark red.

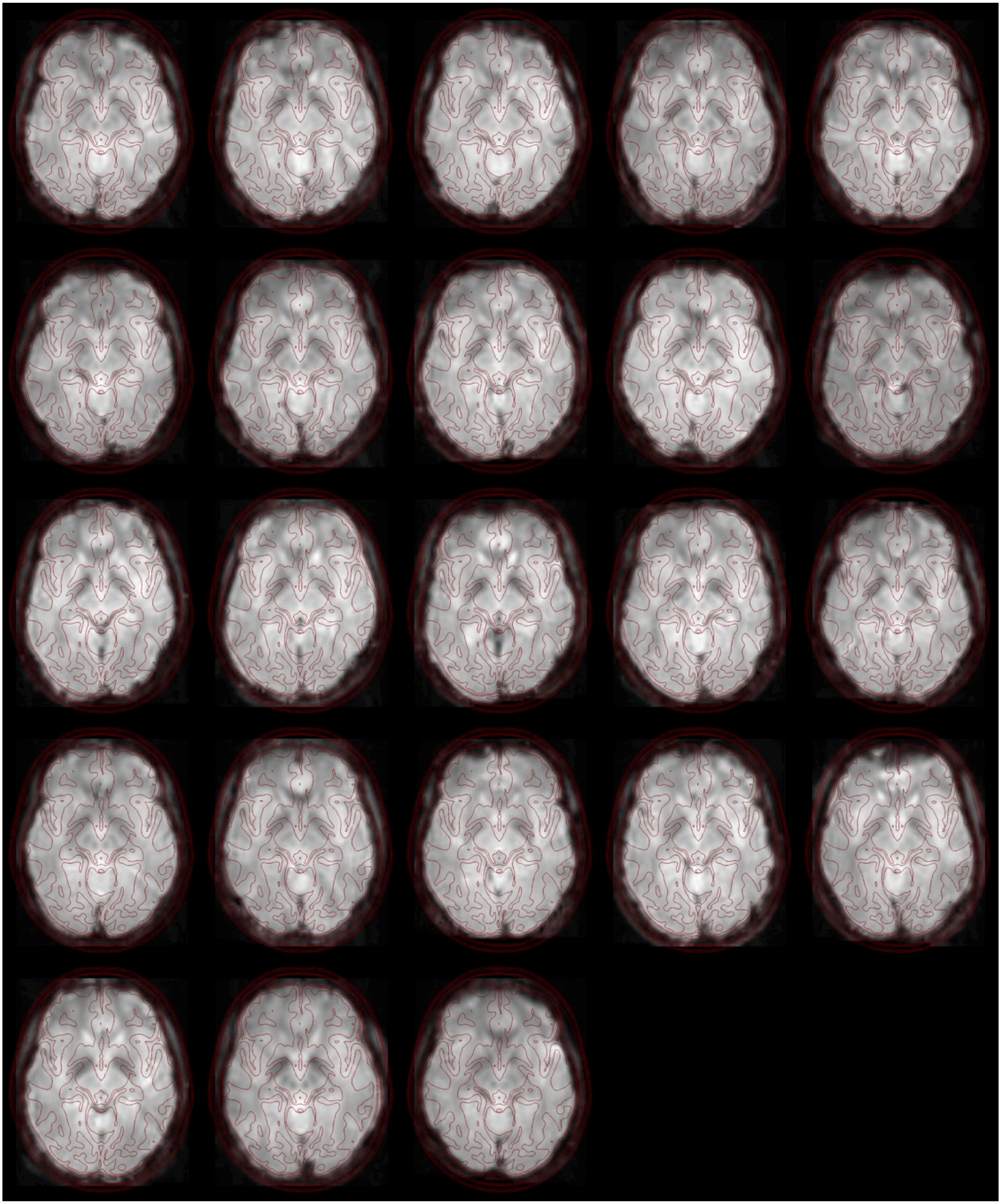

Supplement: Supplementary file 1 — Supplementary Information [file 41598_2017_6105_MOESM1_ESM.pdf]
